# Supplementary material for: Amphibian chytridiomycosis outbreak dynamics are linked with host skin bacterial community structure
Source: Nat Commun. 2018 Feb 15;9:693. doi: 10.1038/s41467-018-02967-w (PMC5814395; doi:10.1038/s41467-018-02967-w)
Supplement: Supplementary file 1 — Supplementary Information [file 41467_2018_2967_MOESM1_ESM.pdf]

SUPPLEMENTARY INFORMATION

Supplementary Note 1: Monitoring long-term infection dynamics of *Batrachochytrium dendrobatidis* (*Bd*) among Pyrenean populations of *Alytes obstetricans*

Bd has been monitored in each *A. obstetricans* population since its initial emergence in Acherito in 2004. Temporal surveys incorporating catch-mark-recapture (CMR) and visual encounter surveys of larvae from each population have shown different trajectories of disease outcome[1]. Population abundance estimates were made based on larvae rather than metamorphs due to the elusiveness of metamorphs. Larvae are also considered a good proxy for metamorph abundance given that larval numbers are a function of adult breeding success and are therefore proportional to the abundance of surviving metamorphs. Our long-term data shows that three populations (Acherito, Lhurs and Puits d'Arious) all experienced severe decline in abundance coinciding with the emergence of *Bd*. However, in recent years these populations have resurged to healthy population abundance levels and persist with *Bd*, indicative of an enzootic disease state. Conversely, Ansabere and Arlet have exhibited sustained population decline without signs of recovery suggestive of a continued epizootic state. Larval population abundance data from visual encounter surveys for 2010-2015 are outlined in Supplementary Table 1. Sampling for *Bd* in *A. obstetricans* metamorphs and subsequent qPCR and data processing was carried out as in the *Methods* section of the main text. Prevalence and infection intensity data for metamorphs for 2010-2015 are presented in Supplementary Figure 1.

SUPPLEMENTARY TABLES

Supplementary Table 1. Visual estimates of larval abundance from 2010 to 2015. +less than 100; ++100-1000; +++ more than 1000.

Year	**ENZOOTIC**			**EPIZOOTIC**	
	Acherito	*Puits d'Arious*	*Lhurs*	*Ansabere*	*Arlet*
2010	+++	+	+++	+++	+++
2011	+++	+	+++	++	+++
2012	+++	++	+++	++	++
2013	+++	++	+++	+	+
2014	+++	++	+++	+	+
2015	+++	++	+++	+	0

Supplementary Table 2. Shared OTUs between larvae, metamorphs and environment for each population.

	Combined	*Acherito*	*Puits d'Arious*	*Lhurs*	*Ansabere*	*Arlet*
Unique to Larvae	3561	1855	1562	1716	1617	NA
Unique to Metamorphs	4115	2418	1301	1623	265	1154
Unique to Environment	920	313	257	213	278	602
Shared by Larvae and Metamorphs	1923	631	469	791	180	NA
Shared by Larvae and Environment	1206	385	255	361	366	NA
Shared by Metamorphs and Environment	1127	326	177	371	108	228
Shared across samples	870	242	142	295	92	NA

Supplementary Table 3. Results of PERMANOVA comparing beta diversity of larvae and metamorphs to environmental samples.

Population	*Comparison*	R^2	*F*	*p value*
Combined	Larva-Environment	0.1325	$F_{(1,81)}$=12.372	0.000999
	Metamorph-Environment	0.0809	$F_{(1,69)}$=6.0735	0.000999
Acherito	Larva-Environment	0.17552	$F_{(1,18)}$=3.8319	0.000999
	Metamorph-Environment	0.15426	$F_{(1,16)}$=2.9184	0.002997
Puits d'Arious	Larva-Environment	0.25355	$F_{(1,19)}$ = 6.4538	0.000999
	Metamorph-Environment	0.22667	$F_{(1,9)}$=2.638	0.004995
Lhurs	Larva-Environment	0.28544	$F_{(1,17)}$=6.7908	0.000999
	Metamorph-Environment	0.1624	$F_{(1,13)}$=2.5205	0.002997
Ansabere	Larva-Environment	0.23975	$F_{(1,17)}$=5.3611	0.000999
	Metamorph-Environment	0.25365	$F_{(1,5)}$=1.6993	0.2637
Arlet	Metamorph-Environment	0.11166	$F_{(1,18)}$=2.2624	0.008991

Supplementary Table 4. Significance of pairwise differences in infection intensity (GE) between populations.

Comparison	*Result*
Ansabere-Acherito	<0.0001
Ansabere-Lhurs	<0.0001
Ansabere-Puits	<0.0001
Arlet-Acherito	<0.0001
Arlet-Lhurs	<0.0001
Arlet-Puits	<0.0001
Acherito-Puits	0.997
Acherito-Lhurs	0.756
Puits-Lhurs	0.959
Ansabere-Arlet	0.995

Supplementary Table 5. KO terms with LDA score ≥ 2.

Enzootic Differential Functional Group	*LDA Score*	*Epizootic Differential Functional Group*	*LDA Score*
ABC.SN.A;NitT/TauT family transport system ATP-binding protein	2.21	APOD; apolipoprotein D and lipocalin family protein	2.05
NitT/TauT family transport system permease protein	2.27	TC.FEV.OM; iron complex outer membrane receptor protein	2.70
whiB1_2_3_4; WhiB family transcriptional regulator, redox-sensing transcriptional regulator	2.11		
putative drug exporter of the RND superfamily	2.19		
iron complex transport system substrate-binding protein	2.23		
ABC.PE.A1; peptide/nickel transport system ATP-binding protein	2.00		
ABC.PE.P; peptide/nickel transport system permease protein	2.10		
ABC.SN.S; NitT/TauT family transport system substrate-binding protein	2.24		
prkC, stkP; eukaryotic-like serine/threonine-protein kinase	2.40		

SUPPLEMENTARY FIGURES

(a)

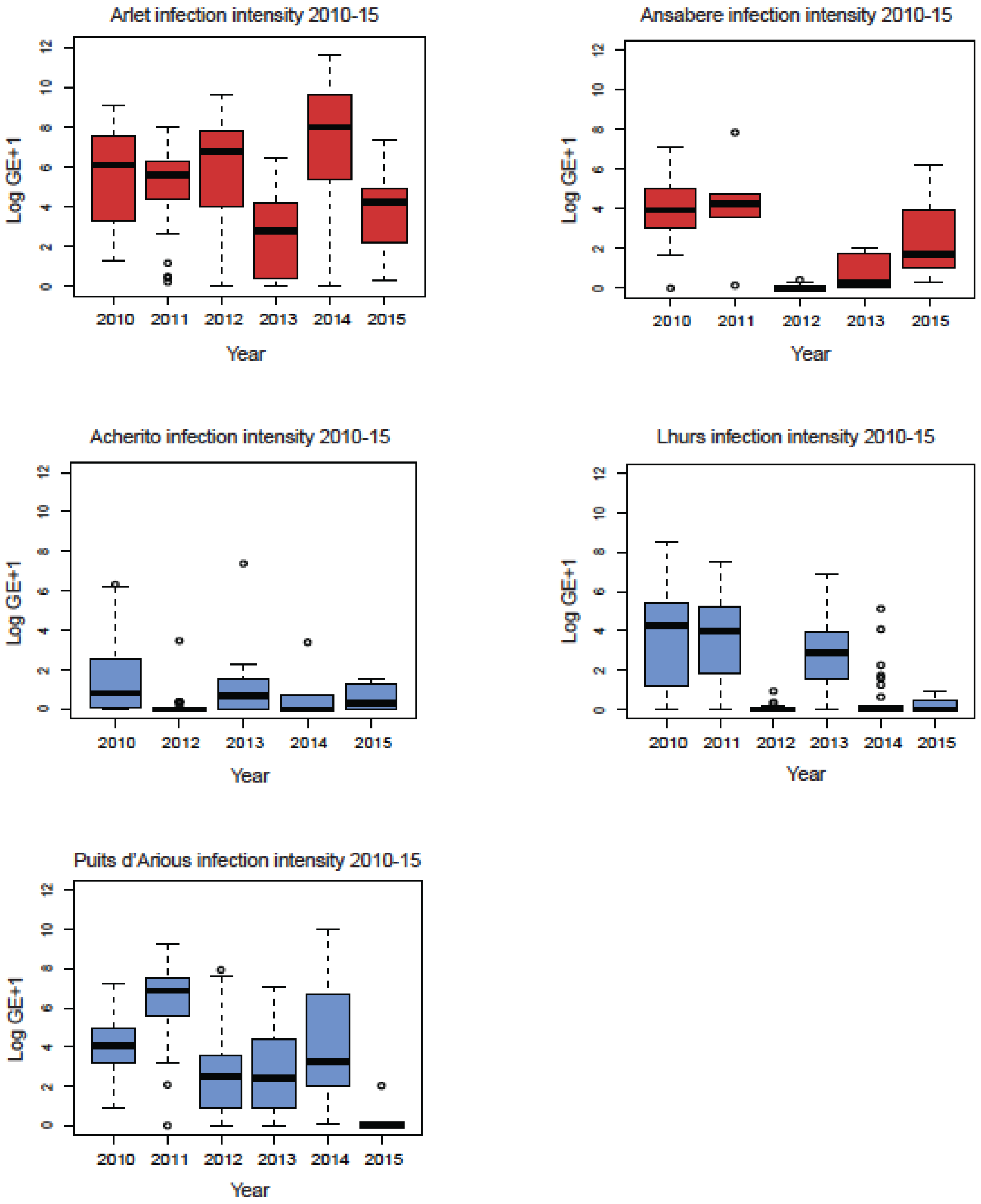

(b)

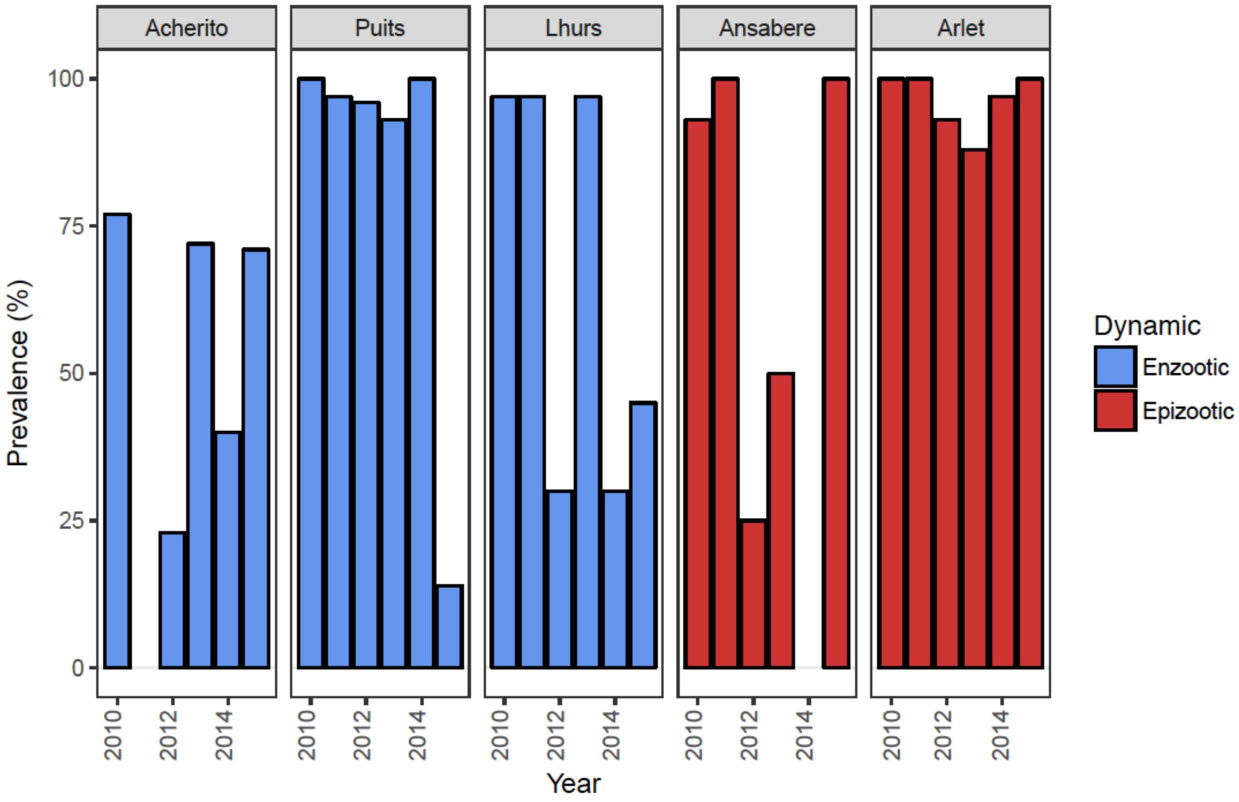

Supplementary Figure 1. (a) Boxplots of metamorph infection intensity (2010-2015) for each population. Boxes represent the 25 and 75 percentile, the horizontal black line is the median, whiskers represent the maximum and minimum values of infection intensity (excluding outliers), open circles are outliers. **(b)** Metamorph *Bd* prevalence (2010-2015).

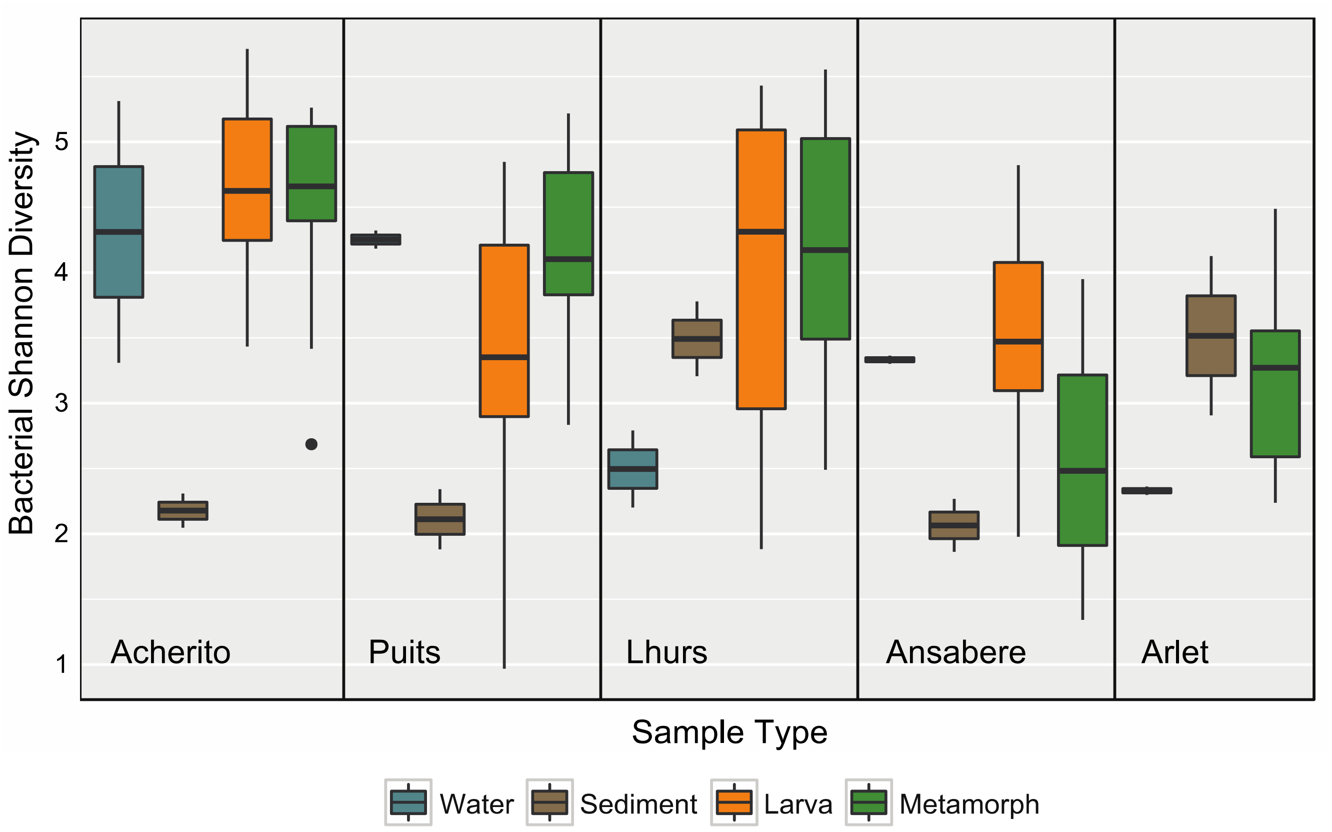

Supplementary Figure 2. Bacterial Shannon diversity by sample type for each population. Boxes represent the 25 and 75 percentile, the horizontal black line is the median, whiskers represent the maximum and minimum values of Shannon diversity.

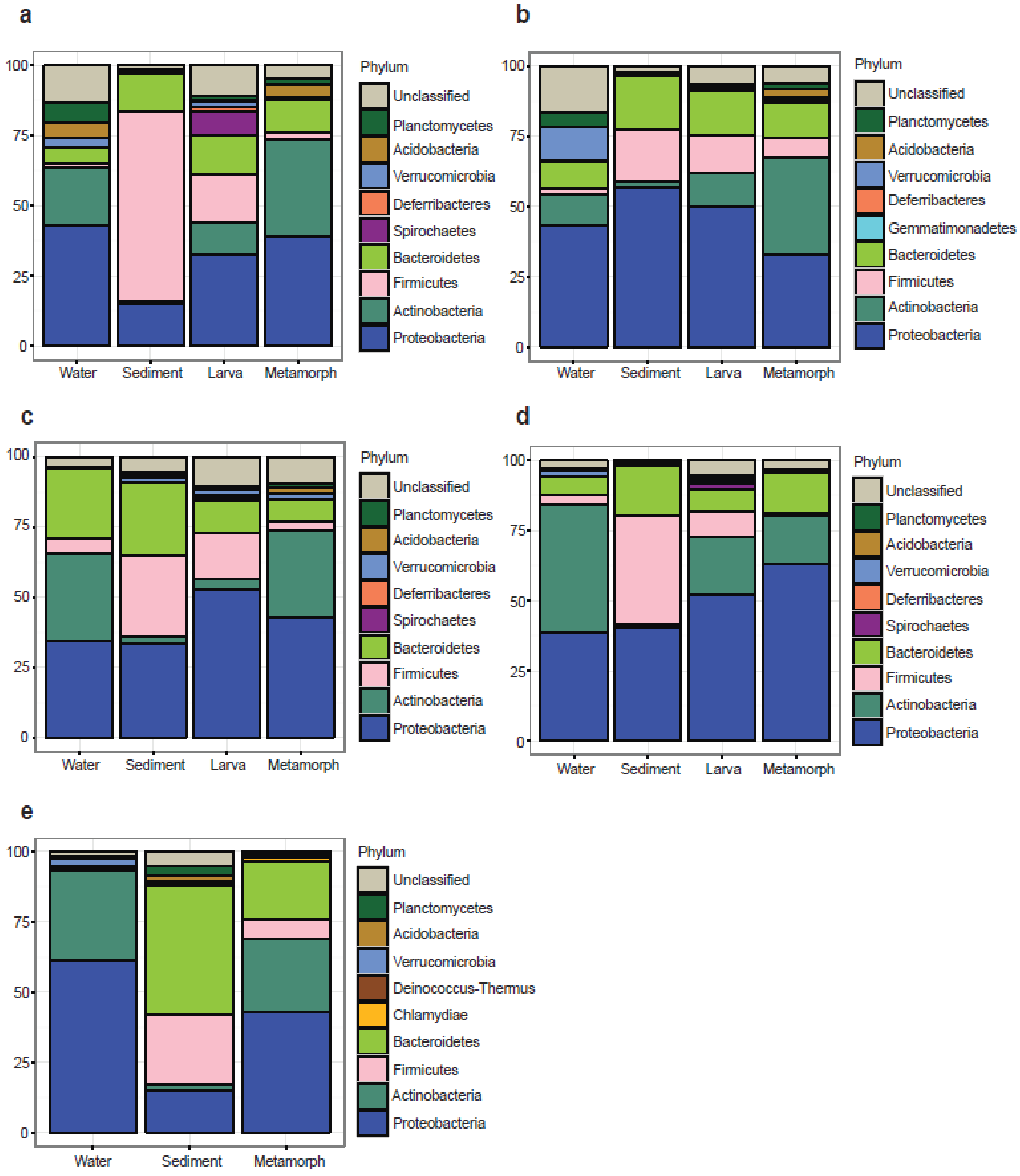

Supplementary Figure 3. Barplots of 10 most abundant phyla for each population by sample type **(a)** Acherito **(b)** Puits d'Arious **(c)** Lhurs **(d)** Ansabere **(e)** Arlet.

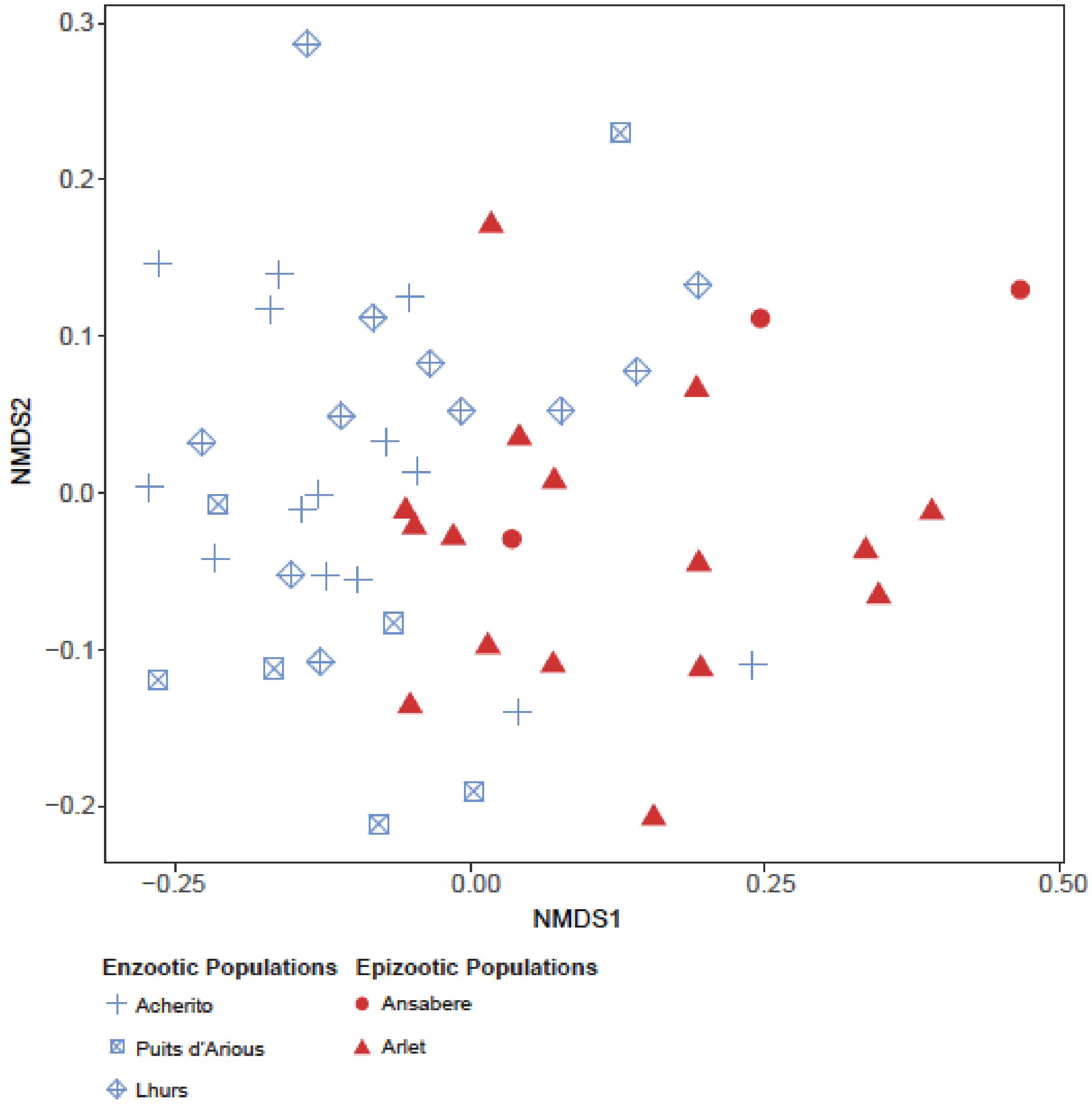

Supplementary Figure 4. NMDS plot based on Bray-Curtis distances displaying clustering of KO functional predictions from metamorph skin bacterial communities from each Pyrenean population.

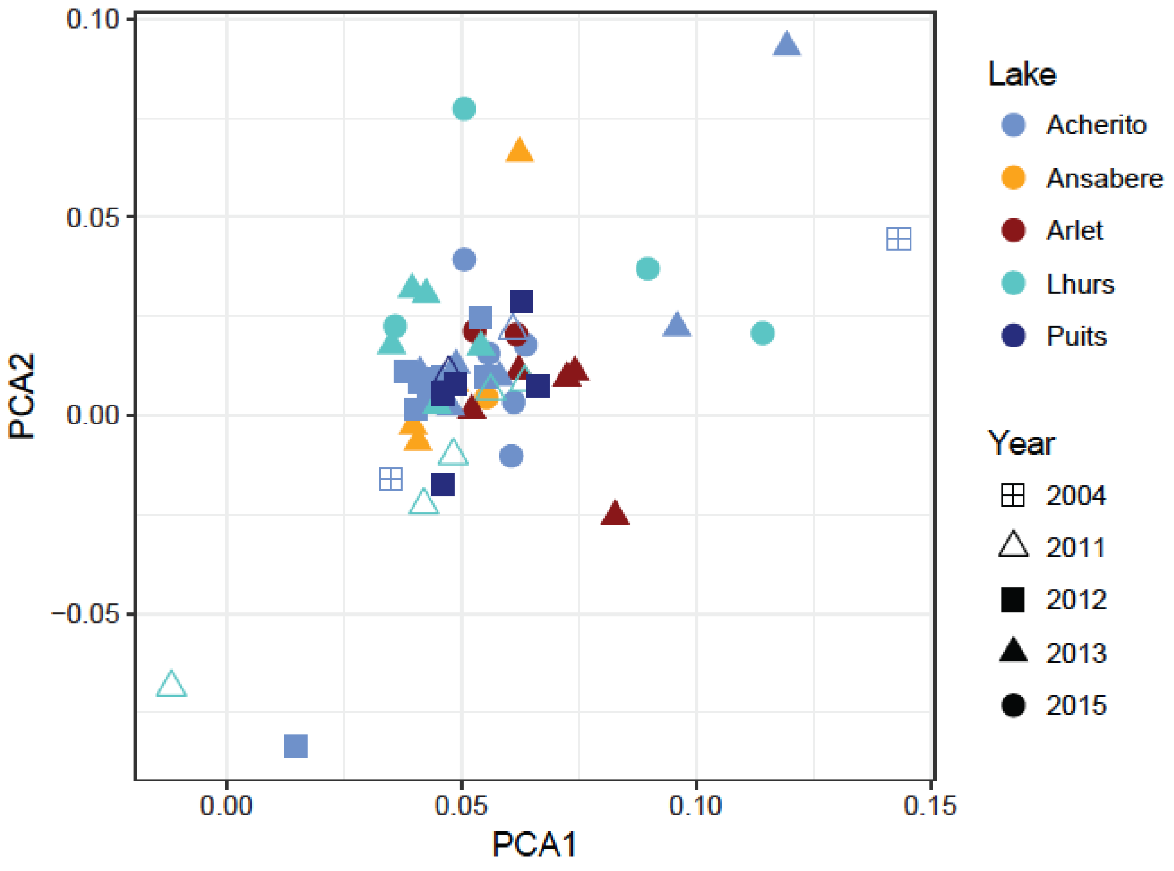

Supplementary Figure 5. PCA plot of SNPs identified from Pyrenean *Bd* isolates by year.

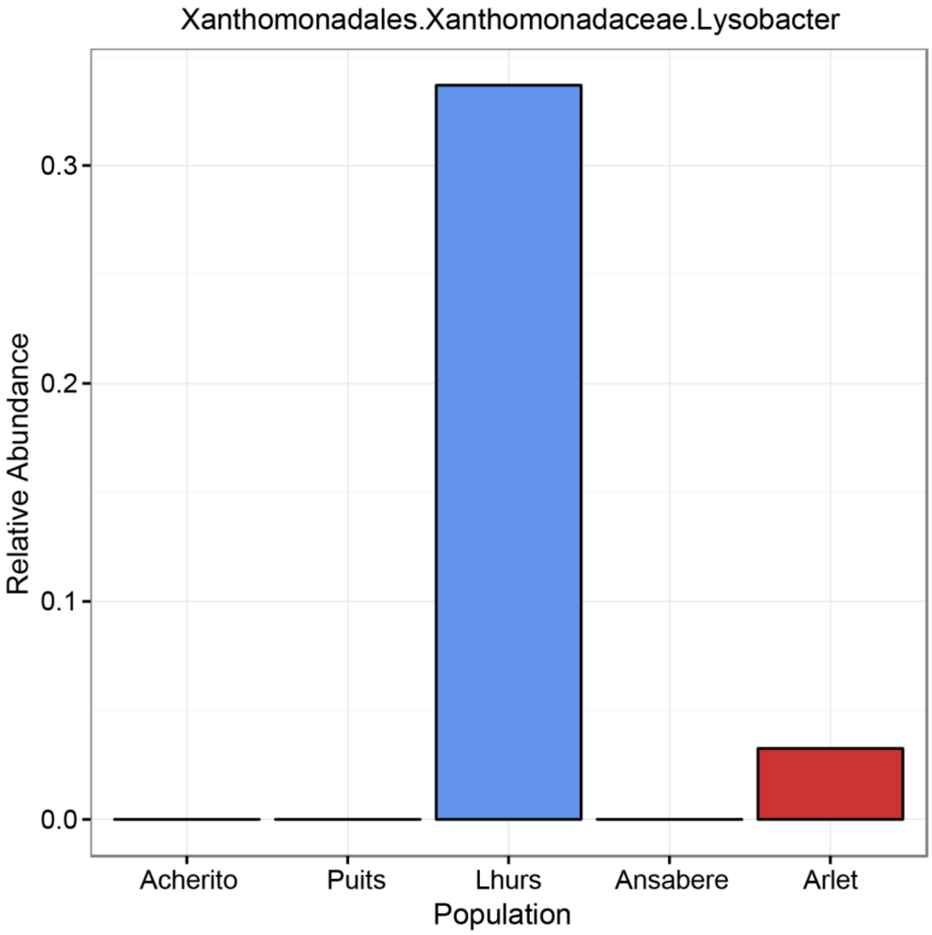

Supplementary Figure 6. Abundance Profile of OTU000483 *Lysobacter* for each metamorph population.

(a)

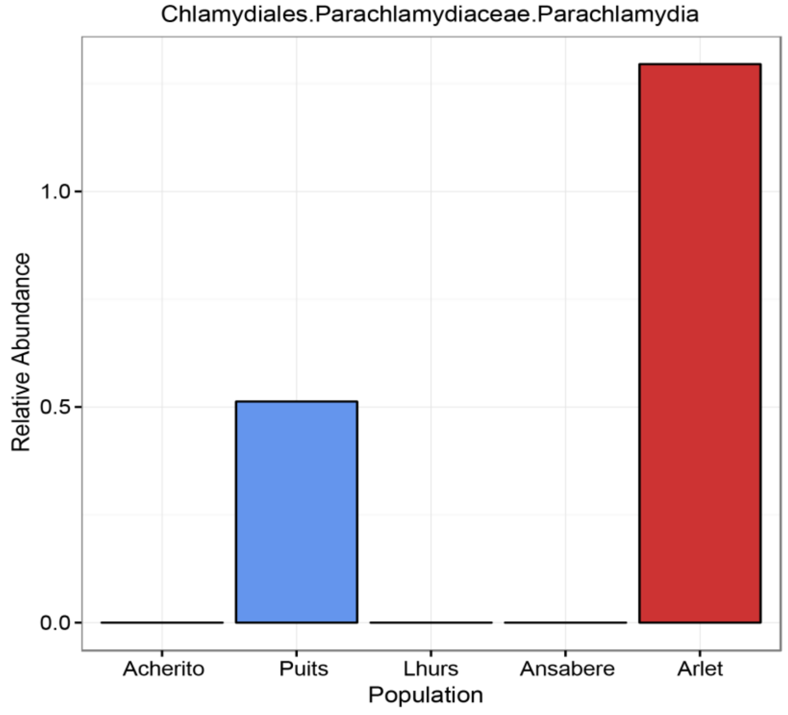

(b)

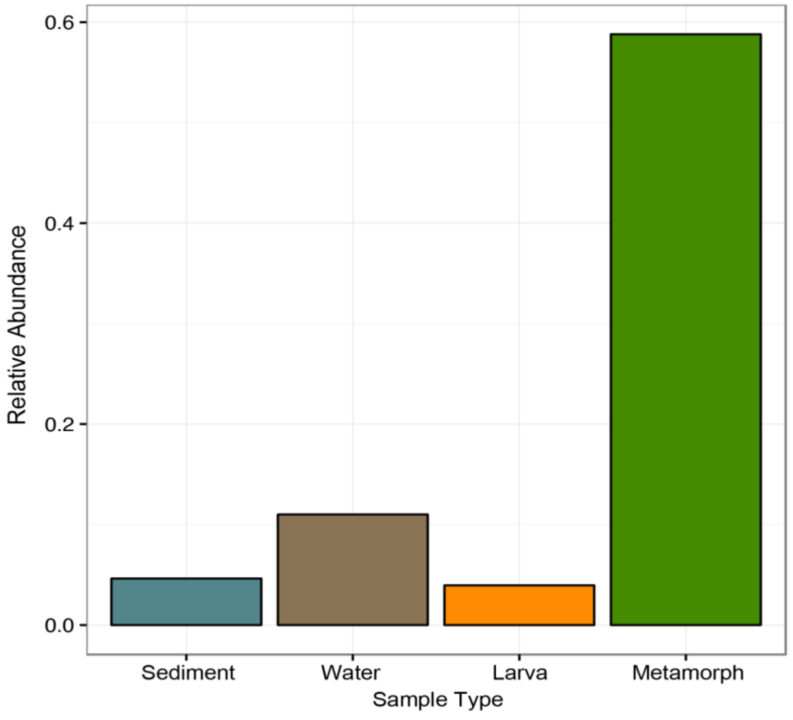

Supplementary Figure 7. (a) Relative abundance profile of OTU000103 *Parachlamydia* for each population **(b)** Relative abundance of Chlamydiae based on sample type for combined populations.

Supplementary References

1. Clare, F.C. *Quantifying the impact of infection by Batrachochytrium dendrobatidis on montane populations of Alytes obstetricans*. PhD thesis, Imperial College London (2014).
