# Supplementary material for: Amphibian chytridiomycosis outbreak dynamics are linked with host skin bacterial community structure
Source: Nat Commun. 2018 Feb 15;9:693. doi: 10.1038/s41467-018-02967-w (PMC5814395; doi:10.1038/s41467-018-02967-w)
Supplement: Supplementary file 3 — Description of Additional Supplementary Files [file 41467_2018_2967_MOESM3_ESM.pdf]

Description of Supplementary Files

File Name: Supplementary Data 1
Description: Excel file of Indicator OTUs of epizootic and enzootic populations.

File Name: Supplementary Data 2
Description: Excel file of Indicator OTUs for each population.

File Name: Supplementary Data 3
Description: Excel file containing summary of *Bd* isolates used for whole genome sequencing.
